# Screening for rheumatic heart disease: quality and agreement of focused cardiac ultrasound by briefly trained health workers

**DOI:** 10.1186/s12872-016-0205-7

**Published:** 2016-02-01

**Authors:** Daniel Engelman, Joseph H. Kado, Bo Reményi, Samantha M. Colquhoun, Jonathan R. Carapetis, Nigel J. Wilson, Susan Donath, Andrew C. Steer

**Affiliations:** Centre for International Child Health, University of Melbourne, Parkville, Victoria Australia; Group A Streptococcal Research, Murdoch Childrens Research Institute, Parkville, Victoria Australia; Royal Children’s Hospital, Parkville, Victoria Australia; Department of Paediatrics, Colonial War Memorial Hospital, Suva, Fiji; College of Medicine, Nursing and Health Sciences, Fiji National University, Suva, Fiji; Royal Darwin Hospital, Tiwi, Northern Territory Australia; Menzies School of Health Research, Casuarina, Northern Territory Australia; Telethon Kids Institute, University of Western Australia, West Perth, Western Australia Australia; Princess Margaret Hospital for Children, Subiaco, Western Australia Australia; Starship Hospital, Auckland, New Zealand; University of Auckland, Auckland, New Zealand; Department of Paediatrics, University of Melbourne, Parkville, Victoria Australia; Clinical Epidemiology and Biostatistics Unit, Murdoch Childrens Research Institute, Parkville, Victoria Australia

**Keywords:** Echocardiography, Cardiac ultrasound, Rheumatic heart disease, Mass screening, Task shifting

## Abstract

**Background:**

Echocardiographic screening for rheumatic heart disease (RHD) has the potential to detect subclinical cases for secondary prevention, but is constrained by inadequate human resources in most settings. Training non-expert health workers to perform focused cardiac ultrasound (FoCUS) may enable screening at a population-level. We aimed to evaluate the quality and agreement of FoCUS for valvular regurgitation by briefly trained health workers.

**Methods:**

Seven nurses participated in an eight week training program in Fiji. Nurses performed FoCUS on 2018 children aged five to 15 years, and assessed any valvular regurgitation. An experienced pediatric cardiologist assessed the quality of ultrasound images and measured any recorded regurgitation. The assessment of the presence of regurgitation and measurement of the longest jet by the nurse and cardiologist was compared, using the Bland-Altman method.

**Results:**

The quality of FoCUS overall was adequate for diagnosis in 96.6 %. There was substantial agreement between the cardiologist and the nurses overall on the presence of mitral regurgitation (κ = 0.75) and aortic regurgitation (κ = 0.61) seen in two views. Measurements of mitral regurgitation by nurses and the cardiologist were similar (mean bias 0.01 cm; 95 % limits of agreement −0.64 to 0.66 cm).

**Conclusions:**

After brief training, health workers with no prior experience in echocardiography can obtain adequate quality images and make a reliable assessment on the presence and extent of valvular regurgitation. Further evaluation of the imaging performance and accuracy of screening by non-expert operators is warranted, as a potential population-level screening strategy in high prevalence settings.

**Electronic supplementary material:**

The online version of this article (doi:10.1186/s12872-016-0205-7) contains supplementary material, which is available to authorized users.

## Background

Rheumatic heart disease (RHD) remains a major cause of morbidity and mortality in resource-poor countries, with an estimated 30 million prevalent cases globally causing in excess of 345,000 deaths annually [1]. The World Health Organization recommends population-based screening for early case detection in high-prevalence areas [2], but very few countries have implemented screening within RHD control programs. Echocardiography has considerably greater accuracy than clinical examination for the diagnosis of mild or asymptomatic RHD [3, 4], and consensus guidelines developed by the World Heart Federation (WHF) [5] allow a standardised definition of ‘definite’ or ‘borderline’ RHD on diagnostic echocardiography. However, the application of these advances in diagnosis to the field of mass screening remains uncertain [6].

A major obstacle to RHD screening at a population-scale is the insufficient number of health workers skilled in echocardiography. Standard training in echocardiography requires considerable time and practice [7, 8] and may not be feasible. An alternate approach to increase capacity may be to develop shorter, focused training programs and reallocate simplified cardiac ultrasound to non-expert health workers, known as task shifting [9]. Task shifting strategies can extend and strengthen available health services [10, 11], yet require operational research prior to introduction to ensure adequate performance and quality [12].

In parallel to considerations of building health workforce capacity, there has been growing acknowledgement of the potential application of focused cardiac ultrasound (FoCUS), rather than standard comprehensive echocardiography, for a range of clinical scenarios, including by briefly trained, non-expert operators [13, 14]. The recent development of international, evidence-based recommendations for FoCUS is therefore highly relevant to RHD screening [15].

In the setting of Fiji, the national capacity to perform echocardiography for RHD is limited to seven physicians and one technologist. Nurses are the largest human resource of the health system (approximately 1 nurse per 500 population) [16], and deliver a range of health services, including school-based health care [11, 17].

In this context, we sought to determine whether shifting FoCUS tasks to the existing school-based nurse workforce was a feasible strategy to implement RHD screening. A pilot study demonstrated that training two nurses to perform basic echocardiography for RHD to facilitate referral was possible, using the identification and measurement of regurgitation jet length as a risk marker of disease [18]. Therefore, we designed a two-part study to further assess this strategy. The first component, presented here, aimed to evaluate the performance of a larger number of non-expert operators in the identification and measurement of valvular regurgitation, after a defined training program. The specific objectives of this study were to evaluate the quality of FoCUS performed by briefly trained nurses; evaluate the inter-rater agreement between these nurses and an expert cardiologist on the presence of valvular regurgitation; and compare the measurements of regurgitation length made by the nurses and cardiologist.

The second component of the study, an investigation of the accuracy (sensitivity and specificity) of screening by non-expert operators, compared with a reference standard diagnosis of RHD, would only be relevant if the quality and agreement were acceptable.

## Methods

### Setting

The study took place in Fiji, a nation of 332 islands in the South Pacific Ocean with a population of approximately 900,000. The Fiji RHD Control Program commenced in 2005. The burden of RHD is among the highest in the world [19], with a prevalence of definite RHD of 8.4 per 1000 children aged 5–14 [20].

### Training

Seven nurses were enrolled in the training program, representing two nurses from each of the major administrative Divisions of Fiji, and one additional nurse to cover the possibility of dropout. None had any previous experience in echocardiography or cardiology. Training was held in Suva, Fiji in in June-July 2012, and has been previously described in detail [21]. The program consisted of one week of classroom-based workshops, run by a pediatric cardiologist and a pediatrician experienced in echocardiography for RHD. This was followed by seven weeks of practical training, where nurses had the opportunity to practice with volunteer children in a school-setting, supervised by an experienced technician. There was no summative or “hurdle” assessment of competency, and as the study aimed to assess this defined training program, no review of images or ongoing training was provided once nurses commenced ultrasound assessment.

### Nurse assessment of regurgitation

**Fig. 1:**
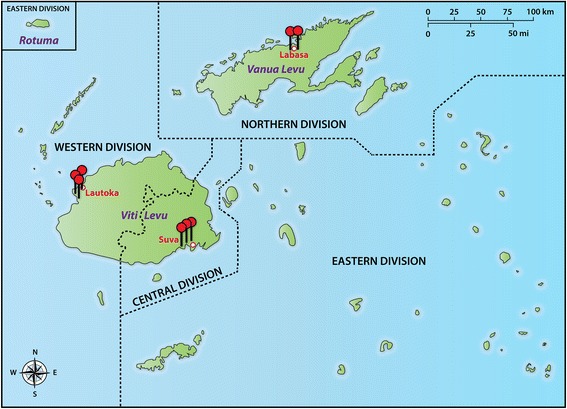
Map of study sites in Fiji. Pins indicate school assessment sites

Nurses performed FoCUS assessments from September 2012 to September 2013 in eight schools, encompassing all three Divisions (Fig. 1). Nurses performed a simplified 12-step protocol focussing on the left side of the heart (Table 1). They were instructed to record the presence of any mitral regurgitation (MR) or aortic regurgitation (AR) on color Doppler imaging in the parasternal long axis, parasternal short axis and apical views, and if present, to measure the longest visible jet in the parasternal long axis and/or apical views. Nurses made assessments and measurements at the time of examination. To minimise the measurement of closing volumes, nurses were instructed to only measure regurgitation seen in ≥ 2 frames. Nurses were not expected to identify morphological changes of the valves.

**Table 1:** Twelve step focused cardiac ultrasound protocol for assessment of regurgitation

View	Valve	Mode	Identify regurgitation	Measure longest jet
1. PLAX	Mitral	2D		
	Mitral	CD	Yes	Yes
	Aortic	2D		
	Aortic	CD	Yes	Yes
2. PSAX	Aortic	2D		
	Aortic	CD	Yes	No
	Mitral	2D		
	Mitral	CD	Yes	No
3. Apical	Mitral	2D		
	Mitral	CD	Yes	Yes
	Aortic	2D		
	Aortic	CD	Yes	Yes

*PLAX* parasternal long-axis view, *PSAX*, parasternal short-axis view, *2D* two-dimensional imaging mode, *CD*, color Doppler mode

The nurses used the M-Turbo portable ultrasound machine (SonoSite Inc., Bothell, WA) which is relatively affordable whilst maintaining adequate quality for assessment of regurgitation on color Doppler. Spectral Doppler is also available but was not used in this study. The machine can run on mains power or battery for several hours, which was a practical advantage over hand-held ultrasound equipment. A 5–1 MHz transducer was used for all studies. Nurses were provided with a simplified manual and taught to operate and adjust relevant settings on the machines. Depth and gain were manually adjusted for each examination. Nyquist limits were set to maximum to avoid overestimation of jet length. All images and loops were saved. Nurses recorded the time examinations commenced (prior to participant lying down) and finished (after saving images and completing data forms). Nurse assessment data were entered into an EpiData electronic database (version 3.1, The EpiData Association, Odense, Denmark).

### Cardiologist reporting

De-identified ultrasounds were sent securely to a pediatric cardiologist for reporting. The cardiologist reported the quality of each echocardiographic view on a scale adapted from similar studies [22] as one of: (1) adequate; (2) poor quality but assessment made; or (3) not interpretable. The categorization was based on an assessment of the completeness of images and loops recorded, orientation, axis and clarity of anatomical structures. Regurgitation present for ≥ 2 frames in any view was noted, and measured if present in the parasternal long axis and/or apical views (i.e., the same assessment as done by the nurse operators). Reports were entered into a REDCap electronic database hosted at the Murdoch Childrens Research Institute [23].

### Participants

Schools were selected from each Division so that each nurse would perform approximately equal numbers of FoCUS examinations. The Fiji Ministry of Education granted permission for research staff to visit each school to explain the procedure to the students, parents, and teachers. Information sheets in Fijian and English were provided to the families of participants. All children aged five to 15 years attending the selected schools were eligible to participate. Signed consent was obtained from the parent or guardian, and assent was additionally required for children aged 10 years and above. Participants also had a standard echocardiogram performed by a sonographer, and were referred to a pediatrician if any abnormalities were suspected. As the seven nurses were also subjects of this study, they gave informed consent prior to participation.

### Statistical analysis

De-identified data were analysed using Stata (version 12, Stata Corp, Texas, USA). The proportion of examinations in each quality category were compared overall, and for each echocardiographic view. Cohen’s kappa (κ) statistic was used to evaluate the inter-rater agreement for the nurses’ and cardiologist’s assessment of the presence of regurgitation in each view, and for regurgitation jet lengths at 0.5 cm intervals up to the WHF diagnostic cut-off point (2 cm for MR, 1 cm for AR). The regurgitation jet length measurements by the nurses and cardiologist were compared using the Bland-Altman method [24]; the difference between length measurements was plotted against the average length measurement, and the 95 % limits of agreement were calculated (mean bias ±1.96 times standard deviations of the bias).

### Sample size

Since the study aim was to compare the measurement of regurgitation length between nurses and cardiologist, the sample size was calculated to ensure a minimum degree of precision for the estimated results. With a sample size of 100 paired measurements, the 95 % confidence interval for each of the Bland-Altman 95 % limits of agreement has a width of 0.34 times the standard deviation of the paired differences [24]. The confidence interval for the overall limits of agreement would therefore be around ~4 % wider than the difference in limits; we considered this to be an adequate degree of precision. We estimated that paired measurements of regurgitation would be available for 15 % (MR) and 5 % (AR) of the sample, and therefore aimed to recruit 2000 children.

### Ethical approval

The study was approved by the Fiji National Health Research Committee, Fiji (Ref 2011 051) and the Human Research Ethics Committee of Northern Territory Department of Health and Menzies School of Health Research, Australia (Ref 11–1649).

## Results

Two thousand eighteen children were enrolled. The number of children assessed by the seven nurses ranged from 239 (11.8 %) to 340 (16.9 %). The mean age was 10.1 years (range 5.1 – 15.7 years) and 51.3 % were female. The ethnicity of participants was 60.9 % iTaukei (indigenous Fijian), 36.6 % Fijians of Indian descent and 2.5 % other ethnicities.

### Quality

The overall quality of nurse FoCUS was adequate in 1949 (96.6 %) examinations; poor but interpretable in 58 (2.9 %) and not interpretable in 11 (0.6 %, Table 2). One nurse had an overall quality (adequate: 81.8 %) that was much lower compared to the other six nurses (adequate: range 97.1 - 99.4 %), and most (48 of 58) of the poor and not interpretable examinations were performed by this nurse.

**Table 2:** Quality of focused cardiac ultrasound by nurses

View	Quality	Nurse 1		Nurse 2		Nurse 3		Nurse 4		Nurse 5		Nurse 6		Nurse 7		Total	
		n = 250		n = 264		n = 340		n = 340		n = 274		n = 311		n = 239		n = 2018	
		n	%	n	%	n	%	n	%	n	%	n	%	n	%	n	%
Overall	1	248	99.2	216	81.8	338	99.4	337	99.1	266	97.1	308	99.0	236	98.7	1949	96.6
	2	2	0.8	42	15.9	0	0.0	2	0.6	7	2.6	3	1.0	2	0.8	58	2.9
	3	0	0.0	6	2.3	2	0.6	1	0.3	1	0.4	0	0.0	1	0.4	11	0.5
MV 2D	1	247	98.8	204	77.3	337	99.1	337	99.1	265	96.7	308	99.0	235	98.3	1933	95.8
	2	3	1.2	59	22.3	1	0.3	3	0.9	9	3.3	3	1.0	4	1.7	82	4.0
	3	0	0.0	1	0.4	2	0.6	0	0.0	0	0.0	0	0.0	0	0.0	3	0.2
MV CD	1	249	99.6	210	79.5	337	99.1	334	98.2	256	93.4	308	97.1	228	95.4	1916	94.9
	2	1	0.4	48	18.2	1	0.3	5	1.5	17	6.2	3	2.9	10	4.2	91	4.5
	3	0	0.0	6	2.3	2	0.6	1	0.3	1	0.4	0	0.0	1	0.4	11	0.5
AV 2D	1	244	97.6	215	81.4	329	96.7	325	95.6	268	97.8	302	99.0	236	98.7	1925	95.4
	2	6	2.4	49	18.6	9	2.7	15	4.4	5	1.8	9	1.0	3	1.3	90	4.5
	3	0	0.0	0	0.0	2	0.6	0	0.0	1	0.4	0	0.0	0	0.0	3	0.1
AV CD	1	248	99.2	224	84.8	338	99.4	340	100.0	266	97.1	308	99.4	236	98.3	1960	97.1
	2	2	0.8	38	14.4	0	0.0	0	0.0	7	2.6	3	0.6	3	1.7	53	2.6
	3	0	0.0	2	0.8	2	0.6	0	0.0	1	0.4	0	0.0	0	0.0	5	0.3

Quality: 1, adequate for diagnosis; 2, poor but diagnosis made; 3, not interpretable. *AV* aortic valve, *MV* mitral valve, *2D* two-dimensional mode, *CD* color Doppler mode

### Agreement of regurgitation assessment

There was substantial agreement between the cardiologist and the nurses overall on the presence of MR (κ = 0.75) and AR (κ = 0.61) seen in two of the three echocardiographic views. The agreement increased when combined with minimum jet length criteria, such that the agreement was highest for MR ≥ 1.5 cm (κ = 0.81) and ≥ 2.0 cm (κ = 0.78); and for AR ≥ 0.5 cm (κ = 0.64) and ≥ 1.0 cm (κ = 0.65). There was moderate agreement on the presence of MR or AR in any one view, and moderate to substantial agreement for individual single views (Table 3). Details of assessment and agreement are shown in Additional file 1: Table S1.

**Table 3:** Agreement between nurse and cardiologist on the presence of regurgitation

View	Jet length (cm)	Mitral kappa (CI)	Aortic kappa (CI)
One view	>0	0.60 (0.56, 0.64)	0.44 (0.32, 0.57)
PLAX	>0	0.72 (0.70, 0.74)	0.54 (0.42, 0.62)
PSAX	>0	0.55 (0.53, 0.57)	0.53 (0.45, 0.61)
Apical	>0	0.61 (0.58, 0.64)	0.44 (0.33, 0.58)
Two views	>0	0.75 (0.71, 0.80)	0.61 (0.46, 0.77)
Two views	0.5	0.76 (0.71, 0.80)	0.64 (0.49, 0.79)
Two views	1.0	0.78 (0.74, 0.83)	0.65 (0.49, 0.81)
Two views	1.5	0.81 (0.76, 0.86)	n/a^a^
Two views	2.0	0.78 (0.71, 0.86)	n/a^a^

*CI* 95 % confidence interval, *PLAX* parasternal long axis, *PSAX* parasternal short axis. Jet length refers to minimum jet length in at least one view. ^a^Analysis was not performed for jet lengths greater than the World Heart Federation diagnostic cut-off point

### Comparison of measurements

**Fig. 2:**
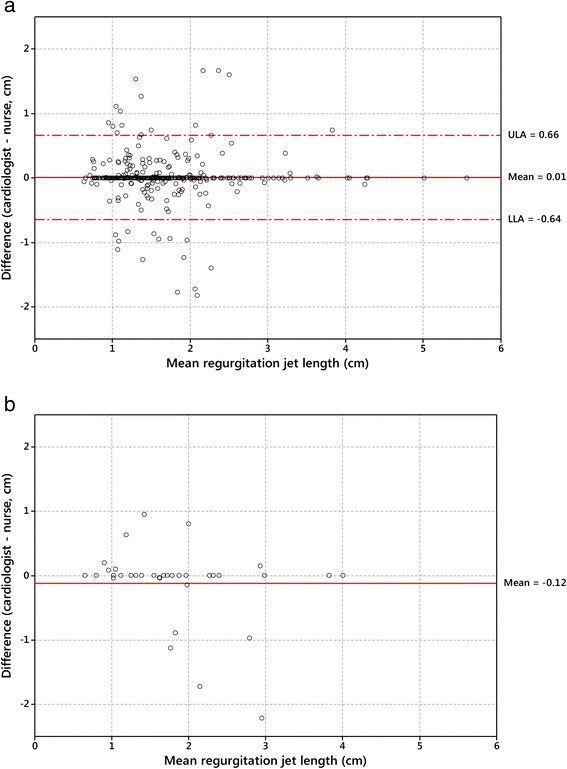
Difference versus mean (Bland-Altman) plots comparing cardiologist and nurse measurements of regurgitation length. **a**: Mitral regurgitation; **b**: Aortic regurgitation. ULA and LLA indicate upper and lower 95 % limits of agreement

There were 442 paired measurements of mitral regurgitation in either the parasternal long axis or apical views, with a mean bias of 0.01 cm and 95 % limits of agreement −0.64 to 0.66 cm (Fig. 2). The number of paired measures for aortic regurgitation (n = 36) was insufficient to calculate the 95 % limits of agreement with confidence, however the mean bias was small (−0.12 cm), most measures were very similar and there were few differences greater than 1 cm. The comparison of measurements in the parasternal long axis and apical views measurements were similar (Additional file 2: Figure S1). In all comparisons, the mean and standard deviation appear uniform through the range of measurements.

### Screening logistics

The median duration for each ultrasound performed by the nurses was 12 min, (interquartile range (IQR), 9 – 17 min) inclusive of saving images and completing all research forms. Each nurse tested a median 11.7 children per day (maximum 30, IQR 8–14).

## Discussion

Our study shows that after completing a brief, structured training program, seven nurses acquired adequate quality FoCUS images in a high proportion of cases. There was moderate to substantial agreement on the presence of regurgitation between nurses and the reporting cardiologist, and measurements of regurgitation jet lengths were similar.

The proportion of ultrasound studies that were considered adequate quality for diagnosis was high, with very few studies (<1 %) deemed inadequate for remote diagnosis. However, the images of one nurse were below the necessary standard, with approximately 20 % inadequate. This variability emphasises the importance of ensuring competency in imaging technique [15], and ongoing quality assurance and monitoring as part of screening program design [25]. As our study was designed to evaluate a pre-defined training program, we intentionally did not incorporate interim review of field images or progressive training. However, we recommend that if non-expert operators are employed for programmatic screening, regular review and ongoing training should be incorporated, which may detect and address inadequacies in specific screening competencies, or with individual operators. Future training procedures could also include a requirement for operators to achieve a minimum standard for image quality. The additional human resource requirements for this ongoing training, supervision and quality assurance should be incorporated into screening program planning. Some variability of imaging performance is to be expected, as the study involved seven individuals. This highlights the inherent variability of workers to perform specific tasks, and the importance of training the most suitable staff for each task.

There was moderate to substantial agreement on the presence of valvular regurgitation. These results are more notable given the conditions: nurses made rapid assessments and measurements at the time of imaging in the field, whereas the cardiologist reported under optimal conditions, including time and the ability to scroll though images and individual frames. Agreement was higher when assessing regurgitation present in two views, which aligns with the definition of pathological regurgitation used in several iterations of RHD diagnostic criteria [5, 26]. Agreement was highest for regurgitation with a minimum jet length at, or 0.5 cm shorter than, the WHF diagnostic cut-off point. These jets are more likely to be clinically significant. The agreement between nurses and cardiologist in our study compare favourably to the agreement between experienced cardiologists (kappa 0.4 – 0.6) on the presence of significant regurgitation reported by Roberts et al. in Australia [27].

**Fig. 3:**
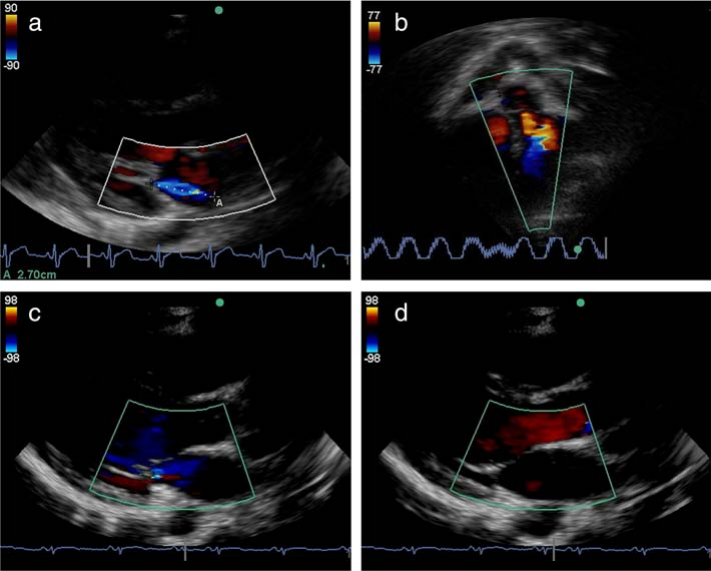
Examples of cardiac ultrasound performed by nurses. **a**. Parasternal long axis view demonstrating mitral regurgitation. This image was categorised adequate quality. **b**. Apical 5 chamber view demonstrating aortic regurgitation. The regurgitation was not identified by the nurse. This image was categorised poor quality. **c** and **d**. Parasternal long axis view demonstrating closing volume. Color is seen across the mitral valve in single frame at the time of valve closure, but not in mid-systole

Despite the overall high agreement, the cases of disagreement warrant further consideration. A large proportion of these jets were short and clinically insignificant, representing physiological regurgitation or closing volumes (Fig. 3). This may be related to the Doppler mode of the ultrasound machine used in the study, which we found to be highly sensitive for detecting closing volumes, usually visible for only one frame and never pansystolic or pandiastolic. Our protocol attempted to allow for this by reporting only multiple-frame jets, although it is possible that the protocol was not followed in all cases, or that some closing volumes were visible in multiple frames. Disagreements for MR included cases where either the cardiologist or the nurse identified a jet, but disagreements for AR appeared to solely represent jets identified by the cardiologist but missed by the nurses. This may indicate the need for further training for the less common finding of AR.

The measurements of regurgitation made by the nurses and the cardiologist were similar in most cases, but there were several large discrepancies. Based on our results, regurgitation length measurements would be expected to be within 1.3 cm for 95 % of individuals assessed. The clinical significance of this result at a population-level is uncertain, however, any discrepancy around the regurgitation length cut-off point may affect the classification between normal or pathological regurgitation in some cases. Overall these findings are encouraging, suggesting non-expert operators with limited training, can not only detect, but also measure regurgitation with adequate reliability in the field. Further studies examining the comparison of measurements, for example, between cardiologists, may help establish the clinical significance of these measurement comparison results.

Our training and evaluation concentrated on valvular regurgitation as a potential risk marker for RHD screening. Four recent publications, using different methods, have also proposed utilising regurgitation as a risk marker [28–31]. However, there are inherent limitations of this approach. Physiological mitral regurgitation is a common and benign finding on echocardiography [32, 33], and therefore measuring regurgitation alone may not differentiate between healthy and diseased individuals. Diagnostic criteria for RHD endeavour to differentiate normal from disease by specifying velocity and spectral envelope requirements for pathological regurgitation and additionally requiring valve morphology abnormalities [5]. However, our experience is that morphological assessment of valve appearance and continuous-wave Doppler are advanced skills beyond the capabilities of non-expert operators (although it may be possible for some workers to acquire these skills with further training and experience.) Further, a screening test using only regurgitation would not differentiate between congenital and rheumatic causes of pathological regurgitation [33], and may miss cases of mitral stenosis and potentially treatable congenital lesions [34]. These factors support the need for diagnostic echocardiography and clinical assessment to determine the final diagnosis for those with abnormal screening tests.

Our study has some limitations. The cardiologist was able to only report on the images saved, and could not determine if a nurse had failed to record a regurgitant jet. This may have resulted in an overestimation of agreement. As each child was assessed by one nurse, and all images reported by a single cardiologist, the assessment of inter-rater agreement between nurses, or between reporting cardiologists was not possible. In addition, there were insufficient cases of AR to accurately calculate the limits of agreement.

The results are representative only of the conditions of our study: the specific training program, the ultrasound machine and protocols, the seven participating nurses and the Fiji school-aged population. Therefore, our findings should be translated with caution to other settings, or using different training programs. The identification and measurement of jets on color Doppler imaging is related to equipment characteristics, and further evaluation using alternate portable or hand-held ultrasound systems is required. In one such recent evaluation, Mirabel and colleagues reported their experience training two nurses using hand-held machines in New Caledonia [30]. After a three-day theoretical component and more than 50 h of highly-supervised practical training, the two nurses obtained good (37-43 %) or fair (56-59 %) images for most patients, and had high sensitivity and specificity (approximately 80 and 90 %) for the detection of RHD compared with a cardiologist assessment. The intensive one-to-one or two-to-one, tailored tutorship used in that study may be more effective for some aspects of imaging, but less feasible in resource-poor settings. Ploutz and colleagues also report high accuracy (sensitivity 74 %, specificity 79 %) of a hand-held screening approach by two nurse operators with some previous echocardiography experience in Uganda [31]. Whilst the results are not directly comparable with our study, both studies support and expand the concept that non-expert operators can learn the skills to perform FoCUS with brief training.

Finally, our research evaluated a human resource strategy to facilitate mass screening in a resource-limited setting, but there are many other important issues pertaining to screening. These include the target population, health system capacity for confirmatory diagnosis and management, the significance of subclinical disease, delivery of effective secondary prophylaxis and cost-effectiveness [35, 36].

## Conclusion

We conclude that after an eight-week training program, nurses were able to obtain adequate quality FoCUS images and reliably assess children in a school setting for the presence and extent of valvular regurgitation. Given these findings, we believe an assessment of the diagnostic accuracy (sensitivity, specificity and predictive values) of screening by non-expert operators, compared to the reference standard (diagnosis by an expert cardiologist) is warranted. Further investigation of the imaging performance of non-expert operators, with variations to the training program, ultrasound equipment and setting is also required, and will inform the overall evaluation of the role of population-based screening in the prevention of RHD.

## Additional files

Additional file 1: Table S1. Details of identification of regurgitation by nurse and cardiologist on nurse ultrasound. (DOC 33 kb) Additional file 2: Figure S1. Difference versus mean (Bland-Altman) plots comparing cardiologist and nurse measurements of regurgitation length in each view of nurse focused cardiac ultrasound. a: Mitral regurgitation in parasternal long axis view; b: Mitral regurgitation in apical 4-chamber view, c: Aortic regurgitation in parasternal long axis view, d: Aortic regurgitation in apical 5-chamber view. ULA and LLA indicate upper and lower 95 % limits of agreement. (JPG 4469 kb)

## Abbreviations

AR: aortic regurgitation
FoCUS: focused cardiac ultrasound
IQR: interquartile range
MR: mitral regurgitation
RHD: rheumatic heart disease
WHF: World Heart Federation
