# Supplementary material for: Screening for rheumatic heart disease: quality and agreement of focused cardiac ultrasound by briefly trained health workers
Source: BMC Cardiovasc Disord. 2016 Feb 1;16:30. doi: 10.1186/s12872-016-0205-7 (PMC4736281; doi:10.1186/s12872-016-0205-7)
Supplement: Additional file 1: Table S1. — Details of identification of regurgitation by nurse and cardiologist on nurse ultrasound. (DOC 33 kb) [file 12872_2016_205_MOESM1_ESM.doc]

#### **Additional Table S1. Details of identification of regurgitation by nurse and cardiologist on nurse ultrasound.**

|  | Regurgitation identified | | | | Agreement, % | Kappa |
| --- | --- | --- | --- | --- | --- | --- |
| View | Nurse and cardiologist | Cardiologist only | Nurse only | Neither |  |  |
| Mitral regurgitation ≥ 1.5 cm seen in two views | 113 | 20 | 28 | 1857 | 97.6 | 0.81 |
| Mitral regurgitation ≥ 2.0 cm seen in two views | 51 | 16 | 11 | 1940 | 98.7 | 0.78 |
| Aortic regurgitation ≥ 0.5 cm seen in two views | 17 | 19 | 0 | 1982 | 99.1 | 0.64 |
| Aortic regurgitation ≥ 1.0 cm seen in two views | 15 | 16 | 0 | 1987 | 99.2 | 0.65 |
