# Supplementary figures and images for: Screening for rheumatic heart disease: quality and agreement of focused cardiac ultrasound by briefly trained health workers
Source: BMC Cardiovasc Disord. 2016 Feb 1;16:30. doi: 10.1186/s12872-016-0205-7 (PMC4736281; doi:10.1186/s12872-016-0205-7)

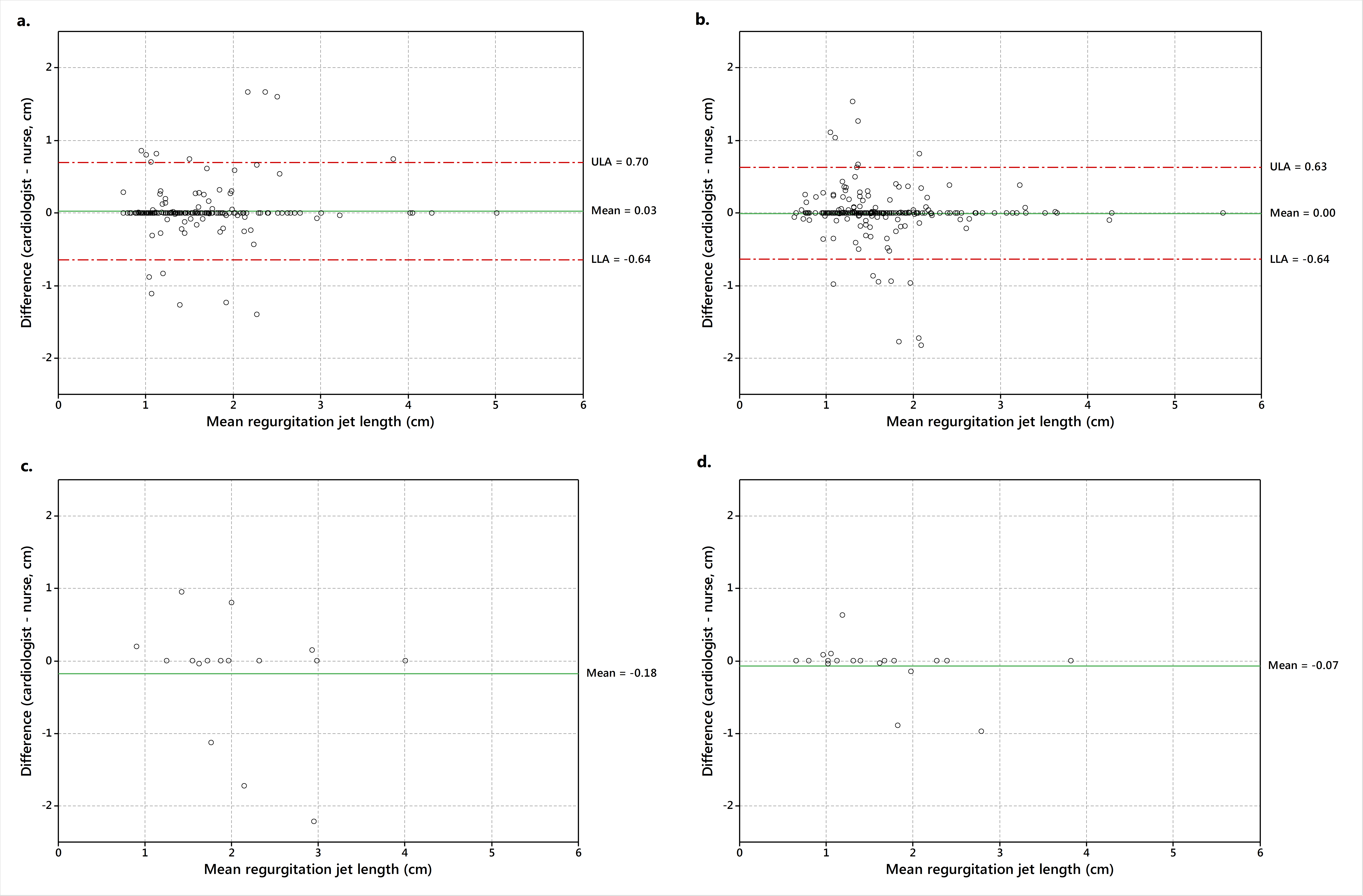

Supplement: Additional file 2: Figure S1. — Difference versus mean (Bland-Altman) plots comparing cardiologist and nurse measurements of regurgitation length in each view of nurse focused cardiac ultrasound. a: Mitral regurgitation in parasternal long axis view; b: Mitral regurgitation in apical 4-chamber view, c: Aortic regurgitation in parasternal long axis view, d: Aortic regurgitation in apical 5-chamber view. ULA and LLA indicate upper and lower 95 % limits of agreement. (JPG 4469 kb) [file 12872_2016_205_MOESM2_ESM.jpg]
